# Trends in Stroke-Related Mortality in the ABC Region, São Paulo, Brazil: An Ecological Study Between 1997 and 2012

**DOI:** 10.2174/1874192401711010111

**Published:** 2017-11-16

**Authors:** Luiz Vinicius de Alcantara Sousa, Laércio da Silva Paiva, Francisco Winter dos Santos Figueiredo, Tabata Cristina do Carmo Almeida, Fernando Rocha Oliveira, Fernando Adami

**Affiliations:** 1 Faculty of Medicine of ABC. Laboratory of Epidemiology and Data Analysis, Department of Collective Health, Av. Lauro Gomes, 2000, Vila Sacadura Cabral, Santo André, SP, Brazil; 2 Faculty of Public Health, University of São Paulo, Department of Epidemiology. Av. Dr. Arnaldo, 715, São Paulo, SP, Brazil

**Keywords:** Stroke-related Mortality, Epidemiology, General mortality, São Paulo, Hemorrhagic stroke

## Abstract

**Background:**

Stroke is the second leading cause of death and the third leading cause of physical disability in the world, with a high burden of morbidity and mortality, but it has been shown a reduction in mortality worldwide over the past two decades, especially in regions with higher income.

**Objective:**

The study analyzed the temporal trend and the factors associated with stroke-related mortality in the cities that make up the ABC region of São Paulo (Santo André, São Bernardo do Campo, São Caetano do Sul, Diadema, Mauá, Ribeirão Pires, and Rio Grande da Serra), in comparison to data from the capital city of São Paulo, in the state of São Paulo, Brazil.

**Method:**

This was an ecological study conducted in 2017 using data from 1997 to 2012. Data were collected in 2017 from the Department of Informatics of the Brazilian Unified National Health System (DATASUS), where the Mortality Information System (SIM/SUS) was accessed. Linear regression analysis was used to estimate the temporal trend of stroke-related mortality according to sex, stroke subtypes, and regions. The confidence level adopted was 95%.

**Results:**

There was a reduction in the mortality rates stratified according to sex, age groups above 15 years, and subtypes of stroke. Mortality from hemorrhagic and non-specified stroke decreased in all regions. However, a significant reduction in ischemic stroke-related mortality was observed only in the ABC region and in Brazil.

**Conclusion:**

The ABC region showed greater mortality due to stroke in males, the age group above 49 years, and non-specified stroke between 1997 and 2012.

## 1 INTRODUCTION

Stroke is among the leading causes of years of life lost due to disability, loss of financial and functional independence, and decreased quality of life. It is the second leading cause of death and the third leading cause of physical incapacitation in the world [1, 2]. Although stroke is a disease with a high morbidity and mortality burden, it has been observed that there has been a reduction in stroke-related mortality worldwide in the last two decades, being higher in regions with higher income [3, 4].

The mortality due to stroke in Brazil, despite presenting rates ​​above those found in Latin countries, also follows the worldwide trend of reduction and is similar to that observed in developed countries [5-7]. This reduction may be influenced by the economic factors of the regions, mainly due to investments in technological advances and treatments that reduce the chance of the individual with stroke to die [8].

In terms of economic development, the southeastern Brazilian region represents one of the main economic hubs of the country, with a special focus on the metropolitan region of São Paulo, which is comprised of the municipality of São Paulo and several adjacent regions [9].

Among these adjacent regions, the cities that constitute the ABC region of São Paulo (Santo André, São Bernardo do Campo, São Caetano do Sul, Diadema, Mauá, Ribeirao Pires and Rio Grande da Serra) played a fundamental role in the process of industrialization and economic development of this metropolitan region. This is illustrated by the gross domestic product (GDP) of the ABC region, ranking fourth behind São Paulo, Rio de Janeiro and Brasília in the national ranking when compared to capital cities [8].

Considering the relationship between economic development and mortality due to stroke, as observed in Brazil, and in the state and municipality of São Paulo, the fact that the region has representative economic power which is similar to that of large Brazilian capitals such as the ABC region of São Paulo, it is unclear whether this economic growth is complemented by a reduction in stroke-related mortality.

The objective of this study was to analyze the temporal trend and factors associated with stroke-related mortality in the ABC region in relation to that observed in São Paulo, the capital city of the state of São Paulo, and in Brazil.

## 2 METHODS

### 2.1 Study Design

This was an ecological study carried out in 2017 using data from 1997 to 2012. This period was chosen due to the change from ICD-9 to ICD-10 in 1996, and due to the update of mortality data being more complete until the year of 2012.

### 2.2 Place of Study

The region of the ABC, São Paulo, Brazil, is comprised of seven municipalities: Santo André, São Bernardo do Campo, São Caetano do Sul, Diadema, Mauá, Ribeirão Pires and Rio Grande da Serra. It has more than 2.7 million inhabitants and a land area of 828 km^2^. In this region, the socioeconomic inequalities are illustrated by the presence of the municipality with the highest Human Development Index (HDI) among the municipalities of Brazil (São Caetano do Sul, HDI: 0.788) and of municipalities with low HDI, such as Rio Grande da Serra (HDI: 0.749) [10, 11] and Rio Grande da Serra (HDI: 0.749) [10, 11].

### 2.3 Data Source

Data were obtained in 2017 from the Department of Informatics of the Brazilian Unified Health System (DATASUS), where the Mortality Information System (SIM/SUS) was accessed.

DATASUS provides health information for states, municipalities and the Federal District. It is a freely accessible database and represents the main source of health information in the country.

The SIM provides data on deaths occurring in Brazil from the notifications emanating from the death certificates, and the causes of these deaths are coded according to the 10th International Classification of Diseases (ICD-10) [12-14].

### 2.4 System Reliability

The reliability of the SIM data can be analyzed by the quality of the information and the territorial coverage of the system. The quality of the system, evaluated by the proportion of deaths reported by poorly defined causes, is approximately 6% [14] and coverage of 96.1% in 2011 [15].

## 3 DATA COLLECTION

### 3.1 Stroke Deaths

For the selection of stroke deaths, the classification by Sacco *et al*. [16], which defines stroke, the following codes was used:

I60 - subarachnoid hemorrhage;
I61 - intracranial hemorrhage;
I63 - cerebral infarction;
I64 - Stroke not specified as ischemic and/or hemorrhagic.

To access the mortality data, the following sequence was performed within the DATASUS system:

- Vital Statistics;
- Mortality between 1996 and 2014, according to 10th International Classification of Diseases (ICD-10);
- General mortality;
- Geographical scope.

In order to perform the collation of deaths by stroke, each of the codes listed above (I60, I61, I63 and I64) was selected separately and in turn, within the item named ICD-10 Category. The deaths related to each code were stratified according to the following variables:

- Sex (male or female);
- Age group (Ranging from 1 to 80 years or more, divided into age groups of every 4 years);
- Region (ABC region, São Paulo municipality, São Paulo state, and Brazil);
- Year (1997 to 2012).

### 3.2 Total Deaths

To access the mortality data, the same sequence previously described for stroke deaths was performed. The first data extraction was with respect to the total deaths for defined causes categorized according to the ICD-10. The categories A00 to U99 were selected; however, the category coded as R00 to R99 was excluded from the total selection.

The second extraction is related to deaths due to poorly defined causes, and corresponds to the data generated from the selection of all ICD-10 codes from R00 to R99, in the ICD-10 Category.

#### Resident population;

The total population was obtained from censuses and intercensal projections, made available by the Brazilian Institute of Geography and Statistics (IBGE) available on the DATASUS website. To access these population data, the following sequence was performed within the DATASUS system:

- Demographic and socioeconomic;
- Resident population;
- Censuses (1980, 1991, 2000 and 2010), score (1996) and intercensal projections (1981 to 2012), according to age group, sex and status of households home situation;
- Geographical scope.

### 3.3 Stroke Deaths

Crude mortality was calculated by the ratio of the number of deaths from stroke and the resident population in the region for each year, and multiplied by 100,000 inhabitants.

StrokeResident population×100.000inhabitants

After estimating the crude rate, the mortality was standardized by age using the direct method, based on the age distribution of the population used by the World Health Organization [17].

Proportional mortality from stroke was calculated by the ratio of total stroke deaths to total deaths from defined causes in the period using the ICD-10 classification codes (A00 to U99, excluding the codes R00 to R99), multiplied by 100.

Total stroke deathsTotal deaths due to defined causes×100

### 3.4 Data Analysis

The subtypes of stroke were analyzed in three groups: hemorrhagic stroke (I60 and I61), ischemic stroke (I63) and non-specified stroke (I64). Age was categorized into three age groups: up to 15 years, 15–49 years, and over 49 years.

Linear regression was used to estimate the temporal trend of stroke-related mortality according to sex, stroke subtypes and regions. The confidence level adopted was 95% and the statistical program used was Data Analysis and Statistical Software for Professionals (Stata) version 11.0^®^.

## 4 RESULTS

**Fig. (1):**
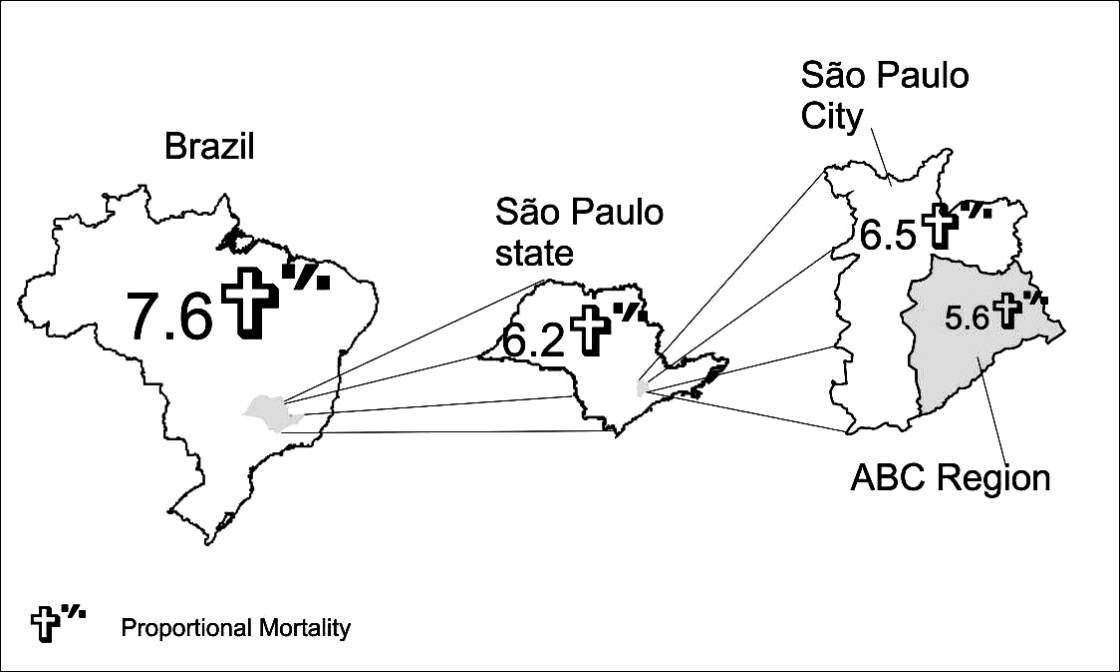
Proportional stroke mortality in the studied regions.

In Brazil, between 1997 and 2012, approximately 8 out of 100 deaths occurred due to stroke. In the state and municipality of São Paulo, approximately 6 out of 100 deaths occurred due to stroke, and in the ABC region, 5 out of 100 deaths (Fig. **1**).

These data represent 1,056,460 stroke deaths in Brazil, 233,792 in the State of São Paulo, 68,257 in the city of São Paulo and 12,228 in the ABC region. In males, the age group above 49 years and non-specified stroke were the characteristics that presented the highest mortality rates in all regions (Table **1**).

**Fig. (2):**
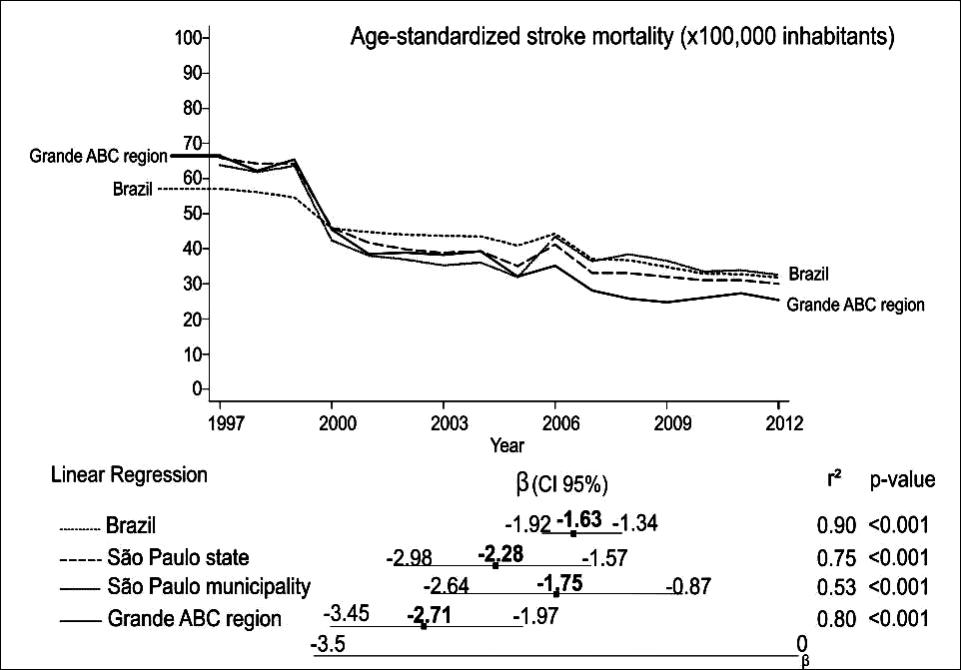
Trends of stroke mortality in regions stratified by age (x100,000 inhabitants).β: regression slope; CI95%: confidence interval; r^2^: predictive capacity.

During this period, a significant reduction in stroke-related mortality was observed in all regions, with emphasis on the difference found between the region of the ABC and Brazil. This difference was estimated by beta confidence intervals varying from -1.34 to -1.92 in Brazil and ranging from -1.97 to -3.45 in the ABC region (Fig. **2**).

The trend of reduction in mortality was also observed when rates were stratified by sex, age groups over 15 years, and stroke subtypes. The hemorrhagic stroke and non-specified stroke subtypes had a reduction in mortality in all regions. However, a significant reduction in ischemic stroke-related mortality was observed only in the ABC region and in Brazil (Table **2**).

## 5 DISCUSSION

This study presents as main findings:

1. Higher mortality rate due to stroke in males, age group above 49 years and non-specified stroke in all study areas (ABC region, city and state of São Paulo and Brazil);
2. Mortality reduction was higher in the ABC region and Brazil, even when rates were stratified by sex, age group and subtype of stroke;
3. The ischemic subtype decreased only in the ABC region and Brazil; the other subtypes decreased in all regions.

In Brazil, there was a decrease in mortality due to stroke over the years, emphasizing that the reduction of social exclusion can be an important factor in reducing the mortality rate due to stroke [18, 19]. This could have also been an important factor in the state and city of São Paulo. However, the mortality rates for cerebral vascular accidents in Brazil are higher than other South American countries, occupying the fourth position in Latin America. The rates in Brazil are higher, even when compared to developed countries [18, 20-22].

Soares *et al*. [23] analyzed the mortality from stroke in large urban centers, such as Rio de Janeiro, Rio Grande do Sul and São Paulo states, and their capitals, Rio de Janeiro, Porto Alegre and São Paulo, respectively, from 1980 to 2006, observing higher stroke-related mortality in men and higher proportional mortality due to stroke in women, similar to that found in our results.

A survey of stroke-related mortality in the city of Brasília during the years 1995 to 2005 also yielded results similar to the present study. Both men and women had a reduction in the mortality due to stroke, and the age group over 80 years had the highest mortality rate when compared with other age groups [24]. It is known that the incidence of stroke increases with increasing age, with double the incidence after 55 years, affecting 19% more men than women [24, 25]. The aging process is also an unmodifiable risk factor that increases the risk of death from stroke [26-28].

However, there is still a difference in mortality among the regions, which may be related to the geographic differences in health coverage within the country, with regions where part of the population is not covered by the distribution of resources intended for public health [29].

The Southeast is notable for having the best specialized centers for early care and assistance to this population [30]. This can be due to investments in advanced healthcare services and in primary healthcare, as a result of which Brazil has become a country with a better life expectancy [31], with representative gains in controlling the risk factors for chronic diseases such as stroke, which benefits from factors such as the reduction in incidence and a more adequate treatment, thus reducing mortality [32].

The decrease in mortality due to stroke during the period studied in the ABC region and in other regions may be associated with improvements in the health service and higher tertiary care which, according to the information technology department of the Unified Health System (SUS), the number of health facilities increased 142% from January 2005 to January 2015 [33, 34].

Therefore, Brazil's improved socioeconomic status, followed by improvements in primary prevention and hospital care, has strongly influenced the decline in the incidence of stroke, and therefore, mortality [9, 35].

### 5.1 Limitation of the Study

It is a fact that there are differences in the social and health spheres between Brazilian regions, and that DATASUS, despite being highly used by the Ministry of Health (MS), has a lack of information on stroke in specific regions, mainly because it does not provide more detailed information on the influence of risk factors on mortality. However, as the main bank of information on Brazilian health, DATASUS can provide the information to improve health policies against stroke. In addition, from 2013 to 2016 we do not have updated data to describe information that would fully meet the objectives of our study.

## CONCLUSION

Stroke is a worldwide problem and still needs special attention to reduce its negative impact on the population. Not only in the way health promotion and treatment should be improved but also, as the population understands and deals with the disease, the information should be more easily accessible, thus giving more focus to the correct completion of the data in DATASUS.

In the period between 1997 and 2012, a reduction in the mortality from stroke was observed. This reduction was greater in the ABC region when compared to the City of São Paulo, State of São Paulo, and Brazil. We have observed also that this reduction was greater in men, the age group above 49 years, and non- specified stroke.

## Figures and Tables

**Table 1:** Description of deaths and mortality in the ABC region, São Paulo municipality, São Paulo state and Brazil, according to sex, age group and subtypes of stroke between 1997 and 2012.

Characteristics	ABC Region		São Paulo Municipality		São Paulo State		Brazil	
	Deaths^†^	Mortality	Deaths^†^	Mortality	Deaths^†^	Mortality	Deaths^†^	Mortality
Sex^#^								
Male	6,140	51.66	32,426	55.52	118,597	55.05	533,634	54.63
Female	6,088	37.91	35,831	40.90	115,195	40.69	522,826	42.53
Age group*								
Up to 15 years	31	0.34	138	0.36	438	0.30	2,155	0.28
15 a 49 years	1,901	8.37	9,742	10.02	30,998	8.89	124,901	8.05
More than 49 years	10,296	157.31	58,377	183.19	202,357	182.06	929,643	193.54
Stroke subtypes^#^								
Hemorrhagic	5,012	15.53	27,335	17.96	80,928	15.09	285,157	12.29
Ischemic	1,060	4.45	13,272	8.79	27,525	5.70	60,470	2.83
NS stroke**	6,156	23.90	27,650	20.20	125,340	26.28	711,072	32.90

† Classified according SACCO *et al*.,^16^ using the codes I60, I61, I63 and I64 of 10th International Classification of Diseases (ICD-10).

^17^^#^ age-standardized.

* Crude rate.

** Not specified as hemorrhagic or ischemic.

ABC Region: The region of the ABC is composed of seven municipalities: Santo André, São Bernardo do Campo, São Caetano, Diadema, Mauá, Ribeirao Pires and Rio Grande da Serra, located in the state of São Paulo, Brazil.

**Table 2:** Linear regression analysis for mortality in the ABC region, São Paulo municipality, São Paulo State and Brazil according to sex, age group and subtypes of stroke between 1997 and 2012.

Characteristics	ABC Region			São Paulo municipality			São Paulo state			Brazil		
	ß (CI95%)	r^2^	p	ß (CI95%)	r^2^	p	ß (CI95%)	r^2^	p	ß (CI95%)	r^2^	p
Sex^#^												
Male	-3.746 (-4.943; -2.550)	0.746	<0.001	-2.218 (-3.512; -0.924)	0.455	0.002	-3.021 (-4.024; -2.019)	0.731	<0.001	-1.942 (-2.307; -1.577)	0.895	<0.001
Female	-2.630 (-3.279; -1.981)	0.832	<0.001	-1.737 (-2.578; -0.896)	0.553	0.001	-2.184 (-2.863; -1.505)	0.756	<0.001	-1.569 (-1.881; -1.257)	0.884	<0.001
Age group												
Up to 15 years	-0.003 (-0.032; 0.025)	0.066	0.794	0.001 (-0.010; 0.013)	0.060	0.795	-0.004 (-0.013; 0.003)	0.029	0.249	-0.002 (-0.007; 0.002)	0.004	0.206
15 to 49 years	-0.428 (-0.544; -0.312)	0.805	<0.001	-0.449 (-0.572; -0.325)	0.799	<0.001	-0.407 (-0.496; -0.317)	0.862	<0.001	-0.321 (-0.359; -0.282)	0.955	<0.001
More than 49 years	-10.036 (-12.867; -7.205)	0.791	<0.001	-6.099 (-10.112; -2.087)	0.391	0.006	-8.963 (-12.023; -5.902)	0.719	<0.001	-6.215 (-7.463; -4.968)	0.882	<0.001
Stroke subtypes^#^												
Hemorrhagic	-0.260 (-0.434; -0.085)	0.381	0.006	-0.557 (-0.695; -0.419)	0.831	<0.001	-0.448 (-0.565; -0.332)	0.817	<0.001	-0.209 (-0.301; -0.117)	0.603	<0.001
Ischemic	-0.754 (-1.309; -0.199)	0.333	0.011	0.168 (-0.527; 0.863)	-0.051	0.612	-0.395 (-0.912; 0.120)	0.101	0.123	-0.178 (-0.315; -0.042)	0.316	0.014
NS stroke*	-2.105 (-2.525; -1.685)	0.884	<0.001	-1.567 (-1.886; -1.248)	0.880	<0.001	-1.727 (-2.069; -1.385)	0.885	<0.001	-1.372 (-1.603; -1.140)	0.914	<0.001

β: regression slope; CI95%: confidence interval; r2: predictive capacity.

^#^ Age-standardized.

† Classified according SACCO *et al*.,^16^ using the codes I60, I61, I63 and I64 of 10th International Classification of Diseases (ICD-10).^17^

* Not specified as hemorrhagic or ischemic.

Source: Mortality Information System (SIM) and Hospital Information System (SIH/SUS). Data made available by the Department of Informatics of the National Health System (DATASUS – www.datasus.gov.br). Ministry of Health, Brasil.

ABC Region: The region of the ABC is composed of seven municipalities: Santo André, São Bernardo do Campo, São Caetano do Sul, Diadema, Mauá, Ribeirao Pires and Rio Grande da Serra, located in the state of São Paulo, Brazil.
